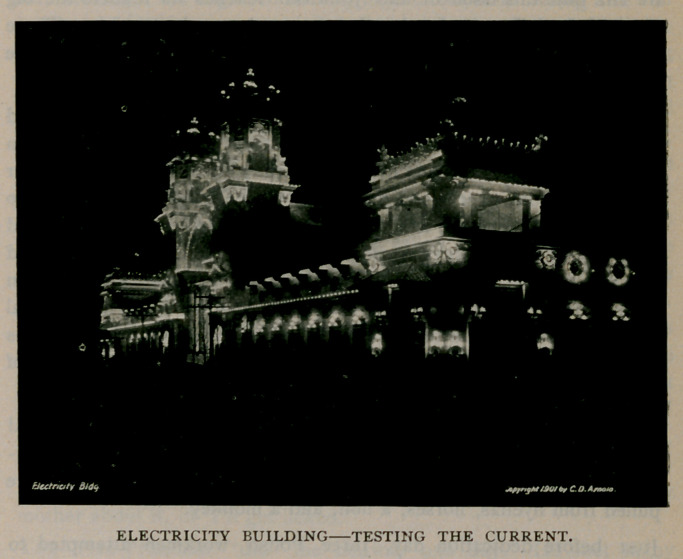# The Pan-Am. “Is” in Buffalo

**Published:** 1901-06

**Authors:** James B. Parke


### PAN-AMERICAN NOTES.

### THE PAN-AM. "IS" IN BUFFALO.

JAMES B. PARKE, Jr., in *Buffalo Evening News*, May 1, 1901.

I am

The great

Pan-Am.,

I am,

And I also am

Here to stay

Through May and

Several other moons,

And

I am the Real Goods and

They can't stop me

Though they've tried by

Hurricanes,

Strikes,

Knockers,

Blockers,

And every other

Old thing, and

Even Uncle Sam

Refused me added increment in the way of

A dinky half-million.

The Pan-American Exposition was formally thrown open to the public on May 1st. Its incomplete condition delayed the formal dedication until May 20th. The ceremonies were brilliant and highly interesting. There were parades, followed by dedicatory ceremonies at the exposition grounds; scientific kite flying, in which great flags, streamers and emblems were sent aloft and floated in mid-air without apparent support, music by combined military bands, ariel bomb explosions setting free Pan-American souvenirs and flags, and speech making and singing at the Temple of Music.

The newspapers have been full of Pan-American news and descriptions, but the most important part of the exposition has not

ELECTRICITY BUILDING—TESTING THE CURRENT.

been exploited to any great extent. The medical department has kept in the back ground, preferring to do its work without any mid-way hurrah or tom-tom beating. The medical director, Dr. Roswell Park, has organised a most perfect hospital service, a handsome building has been erected and occupied and fully equipped. There are five doctors on duty, assisted by six nurses. Emergency cases are taken to the hospital in an automobile ambulance, and after receiving first aid are transferred to one of the city hospitals, the cases being divided among the various institutions. The deputy medical director, Dr. Vertner Kenerson, has charge of the hospital work, and Dr. Nelson W. Wilson is sanitary officer of the exposition.

How well the health of the big fair is safe-guarded may be realised when it is known that during the first days of the exposition Drs. Wilson and Kenerson, under Dr. Park's direction, handled a small epidemic of measles and stamped out the disease without creating a panic. Few people knew that a portion of the midway was under quarantine rule for two weeks.

One of the most interesting exhibits is that of municipal sanitation under the charge of Dr. Jacob S. Otto.

By the time this issue of the Journal reaches its readers the big fair will be fully completed and in smooth running order. There was much delay in opening some of the midway shows, but all are now completed and in running order.

The Indian Congress, where are gathered nearly 300 red men and their squaws and papooses, is one of the most interesting exhibits on the midway. The Indians live here as they do on the plains. Their cooking is done over wood fire in the tepees. Among the Navajo tribe personal hygiene is a strong trait. Daily bathing is a fixed rule. Cold water always is used. Even nursing infants are washed this way summer and winter. A six month's old baby is set in a tin basin and cold water poured over it. Another point of personal hygiene, which has become a tribal characteristic of the Navajo's, is ventilation of clothing. Sleeves are never tight, and that portion of the sleeve covering the armpit is never attached to the garment.

Big Liz, the Bostock elephant, was the star feature of a surgical clinic at the animal show. A spike was cut out of her foot. Following that operation, there was a dental clinic in which teeth were pulled from hyenas, horses, a lion, and a monkey.

Just before dedication day, three Polish workmen attempted to drag an electric light wire out of their way in the machinery Building. One was killed, and the others were badly burned about the hands and arms. Since the foregoing was written another fatal electric wire accident has occurred.

One of the most beautiful sights of dedication day was the flight of 10,000 carrier pigeons, bearing messages announcing the day's event.

The Beautiful Orient is a quaint show. It has brought to the exposition all the light and shadow of Cairo and Constantinople, including their people, their customs, their animals and their smells.

The night view of the exposition is a picture of Fairyland with its sharp shadows, its bright lights and its thousands upon thousands of electric lights shining golden against the inky blackness of the night sky.

Dedication day was singularly free from accidents or mishaps, not to say crime. Even pickpockets fared poorly, thanks to the admirable police detective system, and the watchfulness of the exposition guards.

One boy came near to grief, however, at the first bull-fight of the season, held in the Streets of Mexico. Whitney Devitt, 13 years old, wearing a red high school hat, in excitement at seeing the bull jump a six-foot fence to try his horns on a picador, leaped the inside rail and landed nearly in front of his bullship. The bull rushed at him, but Devitt, "he lay low" and the crooked horns of the bull just missed him in the attempt to gore the boy. The astonished bull halted a moment; a Mexican rough rider dashed into the ring, picked Devitt up by his neck and trousers' seat, tossed him over the rail, and the danger was past.

This is the way the boy himself, describes the affair: "When that little brown bull jumped the first fence and started down the narrow alley toward me he scared me, so I didn't know what I was doing," said Devitt. "I hadn't any business inside the second rail. The first bull they fetched on was so lazy I thought it was all a 'skin game,' but when the little brown bull sent the Mexicans over the fence I wanted to get down closer so I could see it. I didn't think such a runt of a bull could jump over the fence, and when I seen him pikin' for me, I made a scramble the wrong way. Gee, t'was excitin', wasn't it? But I don't want any more little red bulls on my plate. He blew right plum in my ear when he went by. Don't tell my mother about it, or I'll get licked."

The Vice-President "did" the midway one day. He met many acquaintances there; for instance, Bostock, the animal man, is an old friend, we almost said. He showed Col. Roosevelt some fine specimens of mountain lions, and several other kinds of the species. Among other incidents of the visit, the Vice-President christened an Indian baby, born on dedication day.

## Figures and Tables

**Figure f1:**